# Functional Safety Networks and Protocols in the Industrial Internet of Things Era

**DOI:** 10.3390/s21186073

**Published:** 2021-09-10

**Authors:** Giovanni Peserico, Alberto Morato, Federico Tramarin, Stefano Vitturi

**Affiliations:** 1 Department of Information Engineering, University of Padova, 35131 Padova, Italy; giovanni.peserico@phd.unipd.it (G.P.); alberto.morato.3@phd.unipd.it (A.M.); 2 Autec s.r.l., 36030 Caldogno, Italy; 3 C.M.Z. Sistemi Elettronici s.r.l., 31050 Treviso, Italy; 4 Department of Engineering “Enzo Ferrari”, University of Modena and Reggio Emilia, 41125 Modena, Italy; federico.tramarin@unimore.it; 5 National Research Council of Italy, CNR-IEIIT, 35131 Padova, Italy

**Keywords:** functional safety networks, Industry 4.0, Industrial Internet of Things, industrial wireless networks

## Abstract

Functional safety networks are becoming of paramount importance in industrial systems, due to the progressive innovation introduced by the Industry 4.0 paradigm, characterized by high production flexibility, reliability and scalability. In this context, new and challenging applications have emerged such as hyperautomation, which refers to the combination of machine vision, robotics, communication, and learning, with the explicit involvement of humans. This requires the pervasive and ubiquitous connectivity encompassed by the Industrial Internet of Things, typically achieved via wireless systems. As an example, wireless communications are today fundamental to open up to new categories of autonomous devices that can actively collaborate with human personnel in the production process. This challenging scenario has important implications for safety. Indeed, a reliable coordination among sensors, actuators and computing systems is required to provide satisfactory levels of safety, especially in the case of innovative processes and technologies, such as mobile and collaborative robotics. Hence, it becomes imperative to ensure the correct transfer of safety-critical data via communication networks. In this paper, we address the challenges concerned with functional safety networks and protocols in Industrial Internet of Things ecosystems. We first introduce the design characteristics of functional safety networks and discuss the adoption of safety protocols over wireless networks. Then, we specifically address one of such protocols, namely Fail Safety over EtherCAT (FSoE), and provide the results of an extensive experimental session carried out exploiting a prototype system, implemented using commercial devices based on a WiFi network. Finally, the outcomes of the experiments are used as a basis for a discussion about future trends of functional safety in the Industrial Internet of Things era.

## 1. Introduction

The term Functional Safety refers to those (re)active systems able to automatically identify potentially dangerous conditions, thus triggering corrective actions to reduce the level of risk in a system. Part of the overall safety of a system or device is regulated by the the IEC 61508 Functional Safety International Standard series [1]. Functional safety represents a decisive and crucial requirement in several industrial systems, and will play an ever increasing role in the factory of the future, as addressed by the Industry 4.0 paradigm. Indeed, the functional safety global market, including several device types, such as safety sensors/actuators, PLCs, etc., is expected to grow with a Compound Annual Growth Rate (CAGR) greater than 8% until 2025, as reported in [2]. Traditional safety systems, based on dedicated hardware circuits or electromechanical parts, will be replaced and effectively improved by Functional Safety Networks (FSNs), namely communication systems used for the transmission of safety-relevant messages, that are designed to implement distributed functional safety systems [3].

As a matter of fact, industrial automation systems have made extensive use of communication networks to connect their components deployed over (possibly large) distributed plant areas [4]. This trend started roughly at the beginning of the 1990s and progressively enforced over the years, thanks to the improvements achieved by such networks in terms of performance indicators, such as timeliness, reliability, dependability and scalability. Today, this scenario is further revolutionized by the adoption of the Industrial Internet of Things (IIoT) [5,6] where a pervasive communication infrastructure allows connection of cloud systems, controllers, industrial equipment, sensors and actuators to dramatically improve the performance of manufacturing systems in terms of product quality, production efficiency, safety and security [7,8].

From the architectural point of view, FSNs exploit functional safety protocols logically placed on top of the protocol stacks of wired industrial networks. This is the case, for instance, of the ProfiSafe protocol used in Profibus and Profinet, or of the Fail Safe over EtherCAT (FSoE) in EtherCAT systems. FSNs are expected to be ever more deployed and integrated in the factory communication infrastructures, so that plant safety data and information will become part of IIoT ecosystems and, as such, they will be accessed and elaborated using the new services and tools made available in such a context [9]. Notwithstanding, FSNs are required to ensure the transmission of safety-related information among nodes with extremely low error probability and bounded reaction times: as a reference, to achieve a SIL 3 (Safety Integrity Level) as specified by IEC 61508, a FSN has to ensure a residual error rate less than $10^{-9}/$h, which is largely lower than what is typically provided by typical industrial networks. This is needed to cope with the strong requirements imposed by safety applications, such as motion control [10], automotive [11,12] and nuclear power energy plants [13], to mention some.

However, IIoT ecosystems are characterized by ubiquitous connectivity, as well as by high flexibility of the communication infrastructures, that have to be easily reconfigurable to cope with the dynamic changes of the production schedule. These features may be satisfactorily addressed by *wireless* communications. Thus, it is envisaged that in IIoT ecosystems, industrial wireless networks will be ever more deployed. However, the available functional safety protocols have been designed and tested for wired networks, and hence, their suitability for typical wireless-based IIoT applications, for example, collaborative robotics and transportation via automatic guided vehicles [14], is still to be assessed.

Moving from the above considerations, in this paper, we address functional safety networks and protocols in the new IIoT context. Particularly, we focus on the implementation of functional safety protocols over wireless networks. We start introducing, in Section 2, the design approach of FSNs, as specified by the IEC 61784-3 International Standard [15]. Then, in Section 3, we make some general considerations about the challenges concerned with safety protocols over wireless systems. In Section 4, we focus on the behavior of the FSoE protocol on WiFi. Particularly, we provide details about a prototype implementation carried out on commercially available components, along with the outcomes of an extensive experimental session. In Section 5, we discuss the obtained results, make some proposals for performance improving and outline some future developments about the deployment of FSNs in IIoT systems. Finally, Section 6 concludes the paper.

## 2. Functional Safety Networks and Protocols

The most popular Functional Safety Networks are defined by the IEC 61784-3 International Standard [15] with specific reference to the Communication Profile Families (CPFs) introduced by the fieldbus standardization framework.

Table 1 lists the CPFs for which functional safety protocols have been defined along with their commercial names.

The functional safety protocols defined by IEC 61784-3 have been designed assuming the underlying communication system behaves like a *black channel*. With such an approach, the safety nodes are not aware of the channel features, nor of the industrial network they rely on, with the exception of the service primitives necessary for data transmission.

Additional FSNs are available. Two of them, namely OPC UA Safety and CANOpen Safety, are noteworthy. OPC UA Safety [16] has been recently defined in agreement with the guidelines of IEC 61784-3, even though it is not (yet) included in such standard. CANOpen Safety [17] is a popular European Standard, designed with a different approach, referred to as *white channel* from that of IEC 61784-3.

**Figure 1:**
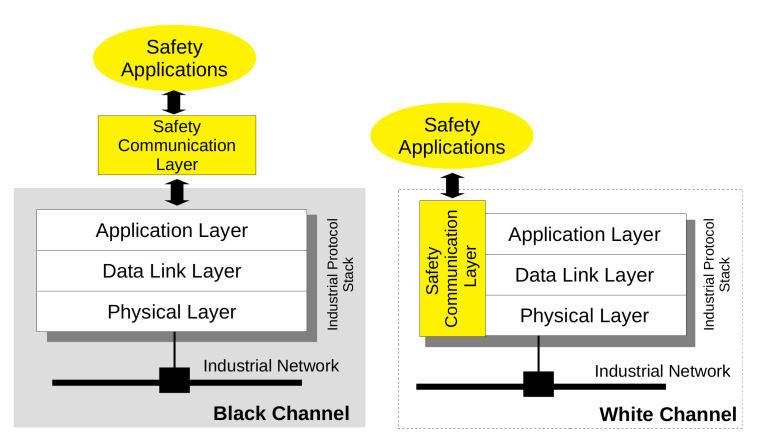
Functional Safety Protocols: Black and White Channel Approach.

A pictorial representation of the two approaches is provided in Figure 1, where on the left side, the communication (black) channel, as seen by the safety communication layer, begins at the boundary with the application layer. With such an approach, although each of the functional safety protocols of IEC 61784-3 has been designed for a specific network, in principle, it could be implemented on whatever network.

Conversely, the white channel approach, as can be seen in Figure 1, right side, assumes that the functional safety protocol is well aware of the underlying communication system and makes use of its services and protocols, in all layers, to implement the safety functions.

Regardless of the channel approach, functional safety protocols have to deal with several types of communication errors. The most typical ones, as specified by IEC 61784-3, are corruption, loss and delay of the transmitted safety messages. To tackle such impairments, the protocols adopt some countermeasures, the most effective being the use of enforced CRC, message numbering and timestamping. The complete list of errors and countermeasures can be found in the standard documents.

When a functional safety network is used (in a plant, machinery, equipment, etc.), it definitely contributes to the risk analysis that has to determine the overall Safety Integrated Level (SIL). In particular, IEC 61508 recommends that the adopted communication facilities may influence with a maximum percentage of 1% the average frequency of dangerous failures per hour of the safety functions (assuming that every single error could lead to a dangerous failure). This reflects on the performance figures that FSNs have to provide, in terms of residual error probability per hour (REP). Clearly, the higher the SIL, the lower the REP. In general, REP is strictly related to the communication behavior and depends on

- The bit error rate of the underlying channel;
- The number of safety messages transmitted per hour;
- The countermeasures against errors adopted by the functional safety protocols.

## 3. Functional Safety Protocol over Wireless Networks

The introduction of the IIoT paradigm relies on a pervasive connectivity that can be ensured by the introduction of wireless networks at all the automation levels.

Hence, the black channel approach discussed so far represents an ideal approach for porting functional safety protocols over those industrial communication systems not having a specific safety extension. In particular, wireless networks could be adopted, paving the way to the introduction and integration of wireless functional safety networks in IIoT ecosystems. However, this clearly represents a challenging issue, since the unreliability of the communication medium may have a negative impact on the behavior of the safety protocols.

Although several analyses and practical experiments have been carried out over the years to address the adoption and the performance of wireless networks in the real-time industrial scenario [4], only some preliminary studies focused on the portability of functional safety protocols on wireless systems. As an example, in [18], the authors refered to WirelessHART and proposed a safety protocol based on an event-triggered technique, called “Safe-WirelessHART”, whereas in [19], the authors presented an implementation of the Fail Safe over EtherCAT (FSoE) protocol over WiFi.

The key aspect, in this context, is represented by the ability of the safety protocols, implemented on wireless networks, to ensure the required performance, particularly in terms of residual error probability and reaction times. Networks of interest are Bluetooth, the IEEE 802.15.4 Wireless PAN, the IEEE 802.11 Wireless LAN and 5G. These are general purpose wireless communication systems that have been (or are going to be, for the case of 5G) considered for industrial applications, possibly in conjunction with purposely developed protocols, and, hence, are good candidates for the black channel approach of functional safety networks.

The residual error probability is a performance index typically adopted by functional safety networks, and hence, unfortunately, no data are available yet for wireless systems. However, REP is strictly related to reliability, for which some considerations can be made. Actually, the aforementioned networks, in their legacy versions, are not suitable for industrial communications, mostly due to the non-negligible percentage of lost packets that may affect their behavior. Nonetheless, the introduction of application layer protocols, specifically conceived for the industrial scenario, brought significant improvements. Some examples in this direction are Wireless HART, which relies on IEEE 802.15.4; the Wireless Interface for Sensors and Actuators (WISA), developed on top of Bluetooth; and Wireless networks for Industrial Automation-Factory Automation (WIA-FA), based on IEEE 802.11. These are able to cope with the stringent reliability requirements of the industrial scenario, revealing performance figures close to those of their wired counterparts. Thus, it may be envisaged that wireless functional safety networks based on the black channel approach may be implemented by replacing the wired industrial network stack in Figure 1 with any of the aforementioned wireless ones.

The reaction time of a functional safety network is defined as the maximum time in which a safety action, triggered by an event, is performed. A typical case is the sending of the emergency stop command to all the safety nodes of the network, as a consequence of a fault detection. In addition, in this scenario, no specific data are available for wireless systems. However, useful indications can be obtained from the performance of industrial wireless networks. Indeed, the most popular FSN protocols (e.g., Profisafe, FSoE and Ethernet Powerlink Safety) are based on a master–slave relationship, where the safety master regularly polls the slaves, exploiting the services provided by the underlying application layer, at the black channel border. Thus, the reaction time of an FSN implemented on top of a given industrial wireless network is strictly related to the polling time of a device connected to that network. If we suppose that the adopted safety protocol is a pure master–slave, $T_{c}$ is the cycle time (i.e., the time necessary to execute the polling of all the slaves), *N* is the number of slaves, and $T_{pi}$ is the polling time of the *i*-th slave, then trivially, the reaction time, $T_{r}$, becomes

(1) $$T_{r}=2T_{c}=2\sum_{i=1}^{N}T_{pi}$$

since one cycle time is the maximum time necessary to detect the fault, and another cycle time, in the worst case, is necessary to notify the command to all the slaves.

As an example, Table 2 reports the indicative values of the polling times for the networks mentioned so far. They have been derived from [20] for WISA, from [21] for Wireless HART and from [22] for WIA-FA. As it is evident, the networks have different performances. However, it has to be stressed that several applications, for example, those concerned with process automation, often do not require very fast reaction times, even if they have to be upper bounded [23,24]. Thus, even slower networks may be profitably used, provided that their behaviors ensure bounded reaction times.

Finally, it has to be pointed out that the addressed networks represent meaningful examples, but further ones may be considered, since the black channel approach does not introduce any specific limitation.

## 4. The Fail Safe over EtherCAT Protocol Implemented on WiFi

In this section, we present an example of implementation of the FSoE protocol on general purpose commercial devices, using WiFi as wireless network. In particular, we provide some details about the testbed and the obtainable performance. A more comprehensive report about this activity is given in [19]. Notably, the proposed implementation does not make use of any industrial application layer, and relies only on the protocol stack of the adopted devices. As a consequence, the implementation cannot take benefit of the techniques typically adopted by the application layer protocols to increase reliability. Thus, from this point of view, the achieved performance can be considered, roughly, as a lower bound for reliability. Conversely, the measurement of the polling time does not include the time necessary to execute the application layer protocol stack, possibly leading to slightly more optimistic results.

FSoE is a Master–Slave protocol, with a unique device referred to as FSoE master, and several FSoE slaves. During normal operation, the master cyclically polls the slaves. The data exchange takes place over FSoE connections that are virtual communication channels established between the FSoE master and each FSoE slave in the initialization phase.

**Figure 2:**
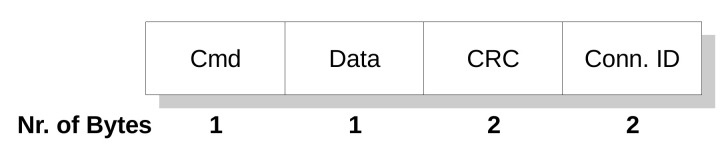
Basic FSoE frame.

The FSoE PDU has two different formats depending on the amount of safe data bytes that has to be exchanged. The simplest format is shown in Figure 2 and it is used to transfer a single byte of safety data from master to slave and vice versa. The first byte contains the command that identifies the specific state of the FSoE connection. This allows to determine the meaning of the safety data. The command field is followed by the data field, by the CRC (2 bytes) and, then, by the Connection Id (2 bytes).

In the following of this section, we provide an experimental assessment of FSoE. Besides the performance indicators discussed in the former section, we address an additional index, namely the Safety Function Response Time (SFRT) which has been introduced for a specific functional safety protocol but may be adopted by FSoE as well.

### 4.1. Safety Function Response Time

SFRT is an interesting index to assess the performance of safety critical automation systems that uses functional safety networks. It represents the time that elapses between the detection of an error and the transition of the system into a safe state. In the context of the IEC 61784–3 International Standard, the SFRT indicator has been specifically introduced by one of the protocols within the specifications, namely ProfiSAFE [25]. In particular, it is defined as “the worst-case time to reach the safe state of the system in the presence of errors or failures in the safety functions or in the communication medium itself”. With respect to the reaction time shown in Equation (1), SFRT represents a more comprehensive index, since it explicitly includes the case of errors in the communication system. Although its specific formulation is related to ProfiSAFE, it has been subsequently characterized by [26,27] for other protocols. In the following, we provide an analytical description of SFRT for FSoE.

In ProfiSAFE, SFRT is calculated under the hypothesis that during the transmission of a safety PDU there may be at most a single faulty device in the safety network. This means that the procedure of reaching the safe state by a system has to consider, in the worst case, also the possibility of a fault in one of the devices of the network. The detection of such fault is made possible by watchdog timers used by the safety devices. Thus, since ProfiSAFE is based on a master–slave protocol, if a slave does not respond to the query of the master within a timeout, then the master marks that slave as faulty and moves to the next one. Conversely, if a slave is not polled by the master within a timeout, then it enters the safe state.

Let us assume the safety network is composed by one master and *n* slave devices. If a slave detects a fault, then the maximum time to reach the safe state is given by

(2) $$SFRT=WCDT+WDTout^{max}$$

where WCDT is the Worst Case Delay Time, i.e., the maximum time requested by the communication between the master and the slaves with the exception of the faulty slave. The contribution of this latter to SFRT is given by the term $WDTout^{max}$, which represents the maximum value of the watchdog timeout among the slaves, formally expressed as

(3) $$WDTout^{max}=\max_{i=1,2,\cdots ,n}\left(WDTout_{i}\right).$$

Moving to FSoE, if a slave detects a fault, in the worst case, it will notify it to the master after a cycle time (this happens when the fault detection occurs just after the slave has been polled by the master and, hence, it has to wait for another cycle before it is polled again). Similarly, another cycle is necessary for the transmission of the safety messages by the master to all the other slaves. However, to calculate the SFRT, it has to be taken into account that one of the slaves can be faulty. Hence, we have

(4) $$SFRT_{FSoE}=T_{C}^{max}+\sum_{\begin{array}{c} i=1\\ i\neq j \end{array}}^{n}T_{p_{i}}^{max}+\max_{i=1,2,\cdots ,n}\left(WDTout_{i}\right)$$

where $T_{C}^{max}$ is the maximum FSoE cycle time, $T_{p_{i}}^{max}$ is the maximum polling time of the *i*-th slave, and *j* accounts for the faulty slave. Clearly, the first two terms in Equation (4) account for the term WCDT in Equation (2).

### 4.2. Implementation

**Figure 3:**
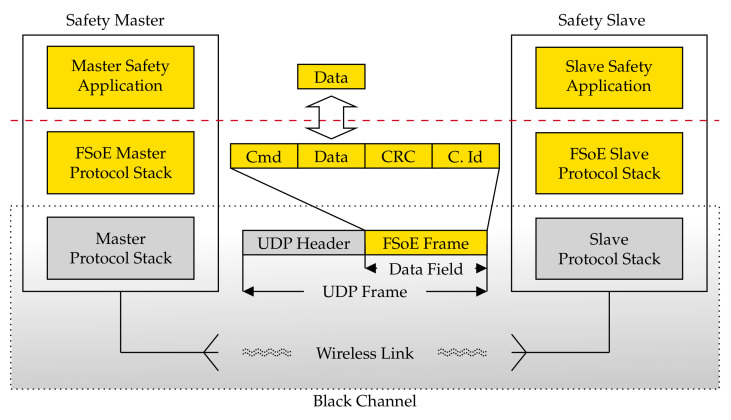
Implementation of the FSoE Protocol Stack.

The FSoE stack has been developed and implemented on two different device types, namely a Raspberry Pi board and a PC running the Linux operating system. As can be seen in Figure 3, the FSoE protocol exploited the User Datagram Protocol (UDP) to carry safety data. With such an approach, as evidenced in Figure 3, the black channel comprises the communication medium, the WiFi modules of the adopted devices, and their TCP/IP suites (indicated as protocol stacks) up to the interface with the respective FSoE stacks.

The transmission of safety data is started by the safety application running on the master that issues a request to the FSoE protocol stack. The safety data are hence encapsulated within the FSoE frame which provides all the countermeasures to detect communication errors. Beyond this point, any further data manipulation/encapsulation is carried out within the black channel. On the slave side, if the integrity of the received safety PDU is positively assessed by the FSoE Protocol Stack, the safety data are forwarded to the slave safety application, which sends the response primitive carrying the response data. The service is then concluded with the arrival of the confirm primitive at the master.

Clearly, a WiFi network has a completely different topology with respect to EtherCAT; thus, a different addressing technique needed to be devised. In practice, the FSoE Master hence assigns a “connection ID” to each slave and associates it with the IP address of the slave itself. This allows the delivery of safety data to the right slave. Remarkably, this custom addressing technique has neither impact on FSoE, i.e., it has not required any modification to the FSoE protocol stack, nor on the safety performance. Moreover, this type of addressing techniques adds a further useful safety feature, because it allows to obtain a double check of the correct addressing of the slave, thanks to the univocal correspondence between IP address and connection ID. Indeed, both slave and master know both their connection ID and IP address. Therefore, upon receiving a frame, they can check whether the addressing is correct or not.

To test the performance of FSoE over WiFi, an experimental setup has been prepared where the FSoE master protocol was implemented on the PC, whereas the FSoE slave was the Raspberry Pi board. The network was configured to use the IEEE 802.11n at a rate of 72 Mbits/s in the 2.4 GHz band with the Request To Send/Clear To Send (RTS/CTS) mechanism enabled. Notably, RTS/CTS is usually disabled, when WiFi is used for industrial real-time applications, since it might introduce additional latency. In this case, however, it has been used since it contributes to increase communication reliability [28].

**Figure 4:**
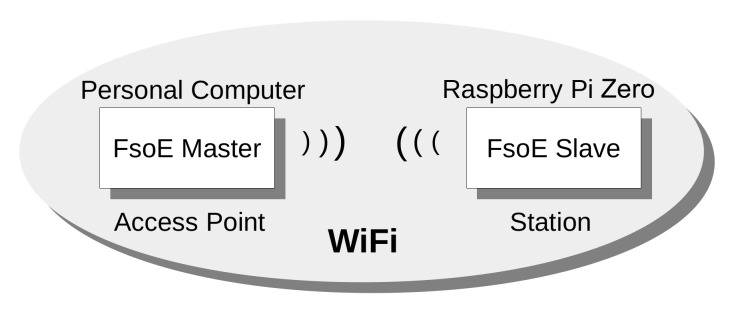
Scheme of the Experimental Setup.

From the IEEE 802.11 network point of view, the node acting as FSoE master is configured as an Access Point (AP), whereas the FSoE slave is a Station (STA), as shown in Figure 4.

### 4.3. Experimental Assessment

A test application has been developed in which the FSoE master application invokes a request primitive to the FSoE master stack to send a safety message to the FSoE slave application and, upon reception of the confirm primitive from this latter one, it immediately starts a new request. These tests were executed in a laboratory environment where in-band interference was controlled. In addition, in this direction, we accurately selected the WiFi channel that showed the lowest traffic. The experiments lasted 5 h. Two meaningful performance indicators were evaluated, namely the percentage of FSoE lost packets and the polling time of the slave.

**Figure 5:**
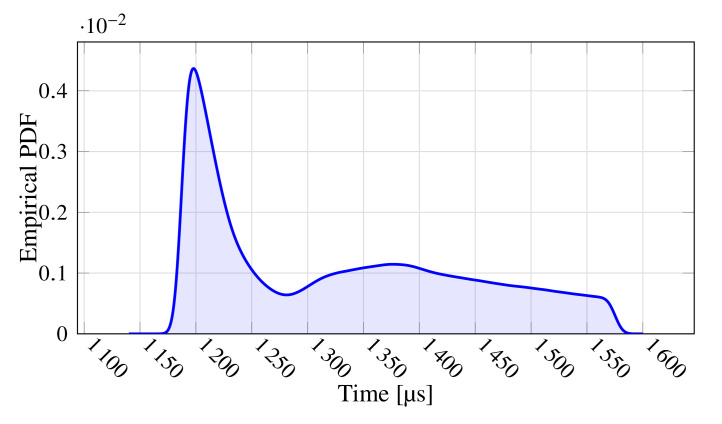
Empirical Probability Density Function of the Polling Time.

During the test, 10 packets were lost in total, resulting in a ratio of lost packets equal to $1.04\times 10^{-6}$. Concerning the polling time, Figure 5 shows its empirical probability density function (to ease readability, the figure reports data comprised in the interval median ± one standard deviation). As can be seen, the behavior is slightly multi-modal with the main peak centered at about 1200 μs. It also denotes a considerable jitter, which might prevent the use of this network in the most demanding applications.

The obtained results, particularly the percentage of lost packets, lead us to conclude that, unfortunately, the SIL3 cannot be reached. Indeed, SIL3 requires that an FSoE connection can be re-initialized at most once every 5 h. Since each lost packet corresponds to one re-initialization, in these experiments, 10 re-initializations occurred.

The calculation of *SFRT* is straightforward from Equation (4). Considering that the watchdog time has been set to 250,000 []μs on both master and slave, it results in

(5) SFRT=17942.40+250,000μs=267.942,40μs

### 4.4. Tests in a Noisier Environment with Additional Transport Layer Protocols

A further assessment of the FSoE protocol over WiFi was carried out in a noisier environment. Specifically, in this case, the tests were conducted in a laboratory with more people and equipment, where there was no control on the sources of interference. Moreover, two additional strategies were considered at the transport layer. The first one was represented by the use of TCP, whereas the second one still uses UDP but with an additional caching layer inserted between the UDP and the FSoE protocol stacks. We refer to it as $UDP_{c}$. It has been introduced to specifically handle both duplication and re-transmission of FSoE frames, as the legacy UDP protocol cannot perform such functions. It is worth observing that $UDP_{c}$ does not imply modifications neither to UDP, nor to FSoE.

The caching layer stores both the incoming SPDUs from the UDP protocol stack and the outgoing ones from the FSoE stack. For every outgoing SPDU, the caching layer expects to receive an incoming frame within a predefined deadline $T_{D}$. In case of packet loss, the deadline expires and the caching layer performs a re-transmission of the outgoing SPDU it previously stored. Notably, the deadline is completely independent on the FSoE watchdog timeout that will continue to monitor autonomously for communication delays. Clearly, to effectively exploit the re-transmission mechanism provided by the caching layer, the following relation must hold

$$T_{p}\leq T_{D}<WDTout$$

The handling of duplicate frames is done by exploiting one of the fundamental features of SPDUs. Indeed, FSoE is designed to ensure that two consecutive frames should differ by at least one bit. This is ensured by the use of consecutive sequence numbers as well of CRC. Due to this characteristic, if two identical frames are received, they are certainly duplicated frames. In this situation, the caching layer detects frame duplication simply comparing the received frame with the previously stored incoming frame. The working principle of the caching layer is summarized in Algorithm 1.
**Algorithm 1:**Caching layer
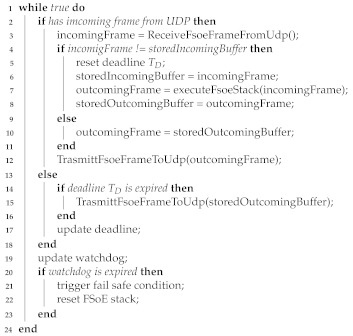


**Figure 6:**
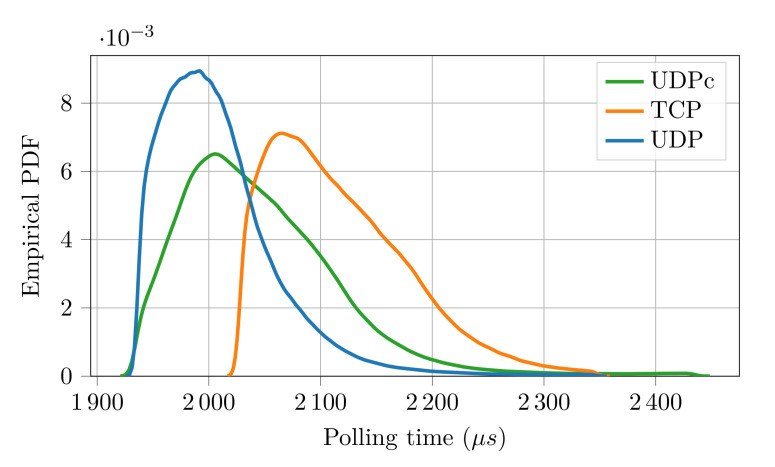
EPDF of the polling time for different transport layers on IEEE802.11.

The results of the experimental campaign are reported in Table 3 (packet loss), Table 4 (polling time statistics) and Figure 6 (polling time empirical PDF).

At a first glance, the (negative) effects of the noisier environment appear evident. Indeed, the performance figures of the protocol when UDP is used are worse than in the previous experiments. Specifically, as can be seen, there were 25 lost packets (15 more with respect to the previous case) whereas the polling time resulted, on average, about 685 []μs longer. However, the two additional strategies considered for the transport layer revealed interestingly better results. As can be seen, with TCP, the number of lost packets was dramatically reduced, however, at the expense of an increased polling time. This is clearly due to the quality of service features introduced by the TCP protocol that ensures a more robust data transmission, but requires longer execution times, since the protocol stack is definitely more complex than that of UDP. Even better performance figures are provided by $UDP_{c}$. In this case, no packets were lost and the polling time was very close to that obtained with UDP. This is because the caching layer introduces only the services necessary to support the FSoE protocol (handling of packet retransmission and duplication), resulting in an efficient stack, with very limited impact on performance.

The SFRT for the three different transport layers are shown in Table 5.

As can be seen, the watchdog timeout has a noticeable impact on the SFRT. Clearly, by decreasing it, the system would result more responsive to communication problems and therefore be able to force the entire system to the safe state much more quickly. However, reducing the watchdog timeout could imply further drawbacks. Indeed, especially when wireless networks are used, the use of a short watchdog timeout might lead to erroneously consider a device as faulty (because it does not answer within the timeout) while, conversely, its polling is simply delayed due to temporary decay of the communication channel status. Thus, the choice of the watchdog timeout is a trade-off between the ability of the system to quickly react to fault situations and the necessity of ensuring the adequate safety performance.

## 5. Discussion and Future Perspectives

The results of the experiments confirm the potential effectiveness of the black channel approach, as they demonstrate the possibility of implementing the FSoE protocol over WiFi and, more generally, over networks for which it was not explicitly designed, without any modification to the protocol itself.

Compliance with the safety requirements imposed by SIL3 could not be demonstrated (actually, for the case of UDP as transport layer, it could not be achieved at all). However, the results obtained with TCP and $UDP_{c}$ are really promising and pave the way to a wide range of applications. These achievements can likely be extended to other protocols that adopt the same approach, e.g., those defined by IEC 61784-3. Among them, ProfiSAFE seems a very suitable candidate, since such a protocol has some common features with FSoE (for example, the master–slave strategy as well as some of the countermeasures against communication impairments). Another safety protocol that could easily work over wireless networks is OPC UA Safety. In this case, the safety stack is implemented (via a suitable mapper sublayer) on top of the OPC UA protocol stack which, in turn, relies on the TCP/IP suite. Thus, the safety protocol is not aware of the underlying physical layer. In this respect, the assessment provided in [29] clearly shows that the use of different physical layers for OPC UA applications may actually have an impact on the performance, but the feasibility of such applications is ensured whatever being the adopted physical layer.

Looking at future applications, 5G cellular networks may be profitably considered for functional safety. Indeed, they are envisaged to be an opportunity for IIoT ecosystems [30]. For example, a 5G-based industrial communication system is presented in [31], where a controller (master) exchanges data with sensors and actuators (slaves) in full-duplex mode. Within 5G, the Ultra-Reliable Low Latency Communication (URLLC) introduced specific features to achieve low latency and higher reliability [32], becoming an interesting candidate not only for typical industrial communication but also for functional safety networks.

Both WiFi and 5G may considerably exploit the Time Sensitive Networking (TSN) set of standards [33], originally conceived for Ethernet networks. Actually, some aspects of TSN are remarkable towards the implementation of functional safety over wireless. Indeed, redundancy and deterministic communication, which are among the key features of TSN, allow to increase reliability as well as to ensure pre-determined reaction times.

Security of industrial networks against cyber attacks [34] is a further important issue that may have a very dangerous impact on functional safety networks, especially when included in IIoT ecosystems. If adequate countermeasures are not undertaken, a threat can compromise the behavior of such networks and, hence, the safety of machinery, equipment and personnel. Unfortunately, most of the functional safety protocols have not built-in security countermeasures, since at the time they were designed security was not an issue. Consequently, they have to rely on the countermeasures implemented by the infrastructure networks they are connected to. A significant exception in this context, however, is represented by OPC UA Safety protocol that actually uses the native OPC UA security features.

Finally, we would like to mention an interesting example of an industrial network that integrates security and safety, provided in [35]. In this paper, the authors describe an implementation of the CANopen protocol on real-time Ethernet networks that includes safety and security mechanisms. Although the resulting architecture is neither compliant with CANopen safety, nor with other safety protocols, its performance figures are satisfactory in that the network provides effective security countermeasures against some types of attacks, as well as safety countermeasures similar to those of the traditional functional safety protocols.

## 6. Conclusions

Functional safety networks are expected to be increasingly used in IIoT ecosystems, particularly over wireless media. In this respect, in this paper, we addressed safety protocols, focusing on those described by the IEC 61784–3 International Standard, and investigated their suitability for IIoT applications. The provided analysis, as well as the results of an extensive experimental session carried out on a prototype implementation of FSoE over WiFi, allowed us to make some interesting considerations. First, the black channel principle can be successfully exploited to bring safety protocols over communication media different from those for which they were natively designed. Second, although the black channel approach in principle ensures feasibility, it is clear it might introduce limitations, particularly with respect to performance. This aspect derives from the undeniable fact that each protocol has been conceived for a specific network. As an example, referring to wired systems, functional safety protocols designed for Ethernet networks can be difficultly implemented on communication systems that use short payloads such as Controller Area Network. Even more evidently, referring to OPC UA Safety, SPDUs may reach large sizes, thus, they need adequate MAC and physical layers to be transferred. Possible performance limitations have been clearly evidenced by the experimental assessment of FSoE over WiFi provided in Section 4. Indeed, as we have seen, the behavior of FSoE is strictly related to the protocol stack included in the black channel. Nevertheless, it has also been shown that there is large room for improvement and future developments. Focusing on WiFi, performance might be further improved with respect to both reliability and reaction time. Indeed, rate adaptation algorithms specifically designed for industrial WiFi applications, as well as suitable network protocol tuning, may lead to significant benefits in terms of reliability and timeliness, as discussed in [36,37]. In addition, new forthcoming WiFi versions, such as those based on the IEEE 802.11ax standard, promise considerable performance improvements. Finally, we remark that the upcoming extension of TSN features to WiFi and 5G systems, thanks to its built-in mechanisms to improve reliability and timeliness, promises considerable performance improvements that could enforce rapid and extensive adoption of safety protocols in IIoT ecosystems.

## Figures and Tables

**Table 1:** Communication Profile Families (CPF) and Functional Safety Protocols defined by IEC 61784-3.

CPF	Commercial Name	Functional Safety Protocol
1	FOUNDATION Fieldbus	FF-SIS
2	Common Industrial Protocol (CIP)	CIP Safety
**3**	PROFIBUS & PROFINET	PROFIsafe
6	INTERBUS	INTERBUS Safety
8	CC-Link	CC-Link Safety
12	EtherCAT	Fail Safe over EtherCAT (FSoE)
13	Ethernet POWERLINK	Ethernet POWERLINK Safety
14	EPA	EPASafety
16	SERCOS	CIP Safety
17	RAPIEnet	RAPIEnet Safety
18	SafetyNET p	SafetyNET p

**Table 2:** Indicative Values of Device Polling Times for Industrial Wireless Networks.

Network	Polling Time
Wireless HART	≈10 ms
WISA	≈1 ms
WIA-FA	≈100 μs

**Table 3:** Lost FSoE Packet Statistics for different transport layers on IEEE802.11.

Packet Loss			
	Total pkt	pkt Lost	% pkt Lost
UDP	7,817,020	25	0.00032
TCP	7,457,630	1	0
UDPc	7,726,440	0	0

**Table 4:** Polling time statistics for different transport layers on IEEE802.11.

Polling Time (μs)				
	mean	std	min	max
UDP	2277.18	1113.25	1183.89	43,128.30
TCP	2366.40	1144.27	1257.90	225,480.00
UDPc	2281.71	1113.14	1187.54	51,522.90

**Table 5:** SFRT values for the three transport layers.

${\mathrm{SFRT}}_{\mathrm{FSoE}}$ [μs]	
UDP	293,128.30
TCP	475,480.00
UDPc	301,522.90

## Data Availability

Not applicable.
